# Fine-Tuning Regulation of Surface Mobility by Acrylate Copolymers and Its Effect on Cell Adhesion and Differentiation

**DOI:** 10.1021/acsabm.2c01053

**Published:** 2023-04-17

**Authors:** Miranda Morata-Martínez, Mark R. Sprott, Carmen M. Antolinos-Turpín, Manuel Salmeron-Sanchez, Gloria Gallego-Ferrer

**Affiliations:** † Centre for Biomaterials and Tissue Engineering (CBIT), Universitat Politècnica de València, Valencia 46022, Spain; ‡ Centre for the Cellular Microenvironment, University of Glasgow, Glasgow G12 8LT, United Kingdom; § Biomedical Research Networking Center on Bioengineering, Biomaterials and Nanomedicine (CIBER-BBN), Valencia 46022, Spain

**Keywords:** acrylate copolymers, substrate mobility, fibronectin fibrillogenesis, cell adhesion, cell differentiation

## Abstract

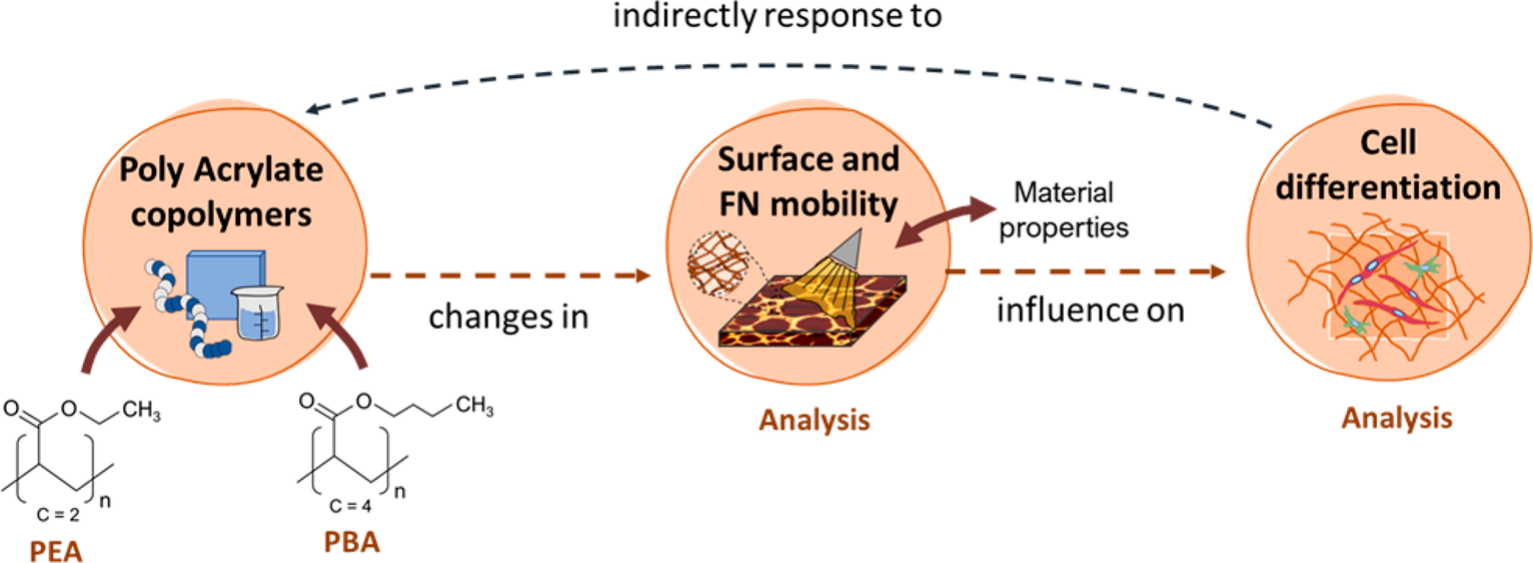

Fibronectin (FN) mediates cell-material interactions during events such as tissue repair, and therefore the biomimetic modeling of this protein *in vitro* benefits regeneration. The nature of the interface is crucial in determining cell adhesion, morphology, and differentiation. Poly(ethyl acrylate) (PEA) spontaneously organizes FN into biological nanonetworks, resulting in exceptional bone regeneration in animal models. Spontaneous network organization of FN is also observed in poly(buthyl acrylate) (PBA) substrates that have higher surface mobility than PEA. C2C12 myoblasts differentiate efficiently on PEA and PBA substrates. In this study, we investigate if intermediate surface mobilities between PEA and PBA induce cell differentiation more efficiently than PEA. A family of P(EA-*co*-BA) copolymers were synthesized in the entire range of compositions to finely tune surface mobility between PEA and PBA. Surface characterization demonstrates that FN mobility steadily increased with the PBA content. All compositions allowed the biological organization of FN with similar exposure of cell binding domains. C2C12 myoblasts adhered well in all the materials, with higher focal adhesions in PEA and PBA. The increase of the interfacial mobility had an impact in cell adhesion by increasing the number of FAs per cell. In addition, cell differentiation decreased proportionally with surface mobility, from PEA to PBA.

## 1 Introduction

Cell-material interactions are mediated by different proteins of the extracellular matrix of tissues, which typically involve cell adhesion proteins such as fibronectin (FN), laminin, vitronectin, and fibrinogen.^1^ When a biomaterial is implanted in the body, a layer of these proteins is deposited on its surface that cells recognize and use to interact with the material. The amount of adsorbed protein, its distribution, and conformation are basic parameters that regulate cell behavior in response to the biomaterial. This interaction is fundamental for cell adhesion and has a determinant role in cell differentiation.^2,3^

Several materials have the ability to induce an extended conformation of FN that allows its assembly into FN networks resembling the physiological process of adhesive cells,^4^ known as material-driven FN fibrillogenesis. Our group has pioneered observing this phenomenon in the polymer poly(ethyl acrylate) (PEA), a well-known biomaterial of the family of acrylates.^5^ We have demonstrated that the extended network of FN on the surface of PEA is very effective in the presentation of growth factor binding domains and cell adhesion sites to cells. In this conformation cell differentiation can be efficiently induced with a very low dose of growth factors presented from the FN network. This is possible due to the synergistic stimulation of integrins and growth factor receptors in cells membranes.^6^ Osteogenic differentiation of mesenchymal stem cells and vascularization of scaffolds based on PEA has been obtained by using a very low dose of BMP-2^6^ and VEGF,^7^ respectively, and outstanding results in bone regeneration have been demonstrated in animal models.^6,8^

When the number of lateral methyl groups of PEA is decreased by one to form poly(methyl acrylate) (PMA), FN adopts a globular conformation onto the polymer surface affecting cell adhesion.^9^ Previous observations concluded that the strength of the FN interaction of FN with PEA is higher than with PMA, leading to a higher molecular mobility of FN on PEA than on PMA, which enhances FN remodelling by cells and improves cell viability compared to PMA.^10^ This finding shows that surface mobility can be a critical parameter to modulate cell responses on biomaterials. For instance, fibroblasts responded to increased molecular mobility of the surface by proportionally decreasing the aspect ratio.^11^ Less-mobile surfaces induced osteogenic differentiation of mesenchymal stem cells, whereas highly mobile surfaces induced adipogenesis,^12^ cardiomyogenesis,^13^ and delayed cellular senescence.^14^ By suppressing the actin filament formation with molecular mobility on the surface, progenitor cells could keep their undifferentiated state.^15^

In polymers, the mobility of the main chains is directly connected with the glass-transition Temperature (*T*_g_). Below it, the main chains are almost frozen and the movements are mainly due to the lateral groups of the chains, which occur at the subnanometer scale. Above *T*_g_, the molecular mobility of the main chains is produced, which comprises distances of several nanometers.^16^ This polymer mobility is translated into a surface mobility that affects the mobility of the proteins adsorbed on the polymer, especially in polymers like PEA that interact strongly with adsorbed FN.^17^ Since cells interact with polymers through the layer of adsorbed proteins using their transmembrane integrins, the mobility of the interface layer of proteins is translated into cell response.

The capacity of PEA in organizing FN into nanonetworks has been found in other materials of the family of acrylates. By the addition of methylene groups in the lateral chain of PEA the homopolymers poly(butyl acrylate) (PBA) and poly(hexyl acrylate) (PHA) were polymerized and their ability to induce FN fibrillogenesis was demonstrated by atomic force microscopy (AFM) images.^18^ The results indicate that the addition of methylene groups in the side chain of PEA without other chemical modification affected the surface mobility of the substrates, which increased with the number of CH_2_ groups. This mobility directly influenced the FN mobility, increasing with the length of the polymers lateral chain and had consequences on cell response.^1,17^ With the increase of the FN mobility its reorganization increased and the differentiation of C2C12 myoblasts decreased.^17^

However, the influence of surface mobility on cell adhesion and differentiation is not necessarily proportional. For example, Kourouklis et al. demonstrated that fibroblasts exhibited nonlinear spreading behavior in response to surface mobility,^19^ proposing a biphasic response of cell area with substrate mobility. Sekiya-Aotama et al.^20^ prepared polyrotaxane surfaces with different mobilities demonstrating that this parameter did not influence the initial adhesion and proliferation of C2C12 myoblasts. When analyzing cell differentiation, they found that intermediate values of surface mobility exhibited the highest expression of differentiation genes. They attributed this effect to the fact that high mobility surfaces inhibit the organization of actin fibers, whereas as the surface mobility was decreased, the organization of actin fibers is promoted up to a maximum level.

Continuing the experiments of our previous work in which PEA and PBA with intermediate values of surface mobility demonstrated the higher levels of differentiation of C2C12,^17^ in the present study we aim to investigate if between these compositions there is an optimal surface to induce cell differentiation. The main novelty of the study resides in exploring if intermediate values of surface mobility between PEA and PBA are optimal for cell differentiation, as the literature demonstrates that the correlation between surface mobility and cell differentiation is not necessarily monotonous.^21^ For this, we prepared a new family of random copolymers of both monomers, P(EA-*co*-BA), by gradually changing their ratios, seeking to regulate surface mobility in an attempt to find a composition that outperforms the pure polymer surfaces to induce myoblast differentiation. Since the surface mobility of PBA is higher than that of PEA, a nonmonotonic dependence of cell differentiation could be obtained for intermediate compositions, the novelty of this study being to find out the effect of intermediate compositions on cell differentiation.

## 2 Materials and Methods

### 2.1 Materials and Surface Preparation

Copolymers were synthesized by radical polymerization of monomer solutions of ethyl acrylate (EA) and butyl acrylate (BA) (Sigma-Aldrich) using 1 wt % benzoin (98% pure, Scharlab) as a photoinitiator. Bulk polymer sheets (ca. 1 mm of thickness) were prepared in the whole composition range by mixing EA/BA in proportions 100/0, 70/30, 50/50, 30/70 and 0/100 (% vol.). Polymerization took place during 24 h under ultraviolet light. Copolymer sheets were dried under vacuum to constant weight at 60 °C to remove unreacted residual monomer.

Thin copolymer films were used for protein coating and cell culture assays. To produce them, bulk materials were dissolved in toluene, at 4% (w/v) for PEA and 6% (w/v) for the rest of the compositions, and subsequently spin-coated. Glass coverslips of 12 mm diameter were cleaned with ethanol by sonication and dried at 60 °C before use. The spin-coating process (Laurell Technologies, USA) was performed by adding 100 μL of the polymer solution on the coverslip and spinning at 3000 rpm for 30 s. The samples were then dried under a vacuum at 60 °C to remove the residual solvent.

Vacuum dried copolymer surfaces were coated with fibronectin (FN, from human plasma, Sigma-Aldrich) from a solution at 20 μg/mL in Dulbecco’s Phosphate Saline Buffer (DPBS), freshly prepared without agitation. A droplet of 200 μL of the FN solution was deposited on the coverslips containing the copolymers, and let the protein adsorb for 1 h at room temperature, to ensure equilibrium. Then, the samples were washed twice with DPBS and dried with nitrogen flow before use.

### 2.2 Copolymer Characterization

Glass transition temperature (*T*_g_) values of bulk samples (ca. 5 mg) were obtained in a differential scanning calorimeter (DSC) 8000 from PerkinElmer. After removing the thermal history of the samples by a first heating scan, the samples were subjected to a second heating scan from −80 to 60 °C, both at 20 °C/min. Nitrogen was used as purge gas through the DSC cell with a flow rate of 20 mL/min. Glass transition temperatures were calculated as the midpoint of the change in the specific heat capacity in the heat flow versus temperature graph. To check any existence of weight loss, samples were measured before and after the scanning.

Young’s moduli at uniaxial extension were calculated by statically deforming bulk prismatic specimens (of ca. 25 × 10 × 1 mm) at room temperature (25 °C) in a SCM3000 Microtest machine. The samples were deformed up to 1.6 times their original length. Young’s modulus (*E*) was calculated as the slope of the initial elastic region in the tension vs deformation graph. Tension speed was set at 10 mm/min, with an acquisition time of 2 s and a maximum force value of 15 N. Five specimens per composition were measured.

Height and phase images (size 1 μm × 1 μm) were obtained from spin-coated films, before and after the FN coating, in an Atomic Force Microscope (AFM) (Nanowizard 3 Bioscience AFM, JPK) operating in AC mode. Images were acquired using silicon cantilevers with pyramidal tip with a resonance frequency of ∼75 kHz. To ensure similar topography profiles of the substrates, root-mean-square (RMS) roughness was calculated from the mentioned height images using the roughness subroutine in the JPK software. Fractal dimension (FD) of the FN networks images (phase) was calculated through the box-counting method (BCM), using box sizes of 2, 3, 4, 6, 8, 12, 16, 32, and 64 pixels, by using FracLac and ImageJ software.

Surface hydrophilicity was analyzed, before and after the FN coating, by water contact angle (WCA) measurements using a Theta optical tensiometer (Biolin Scientific) and employing the sessile drop method. Surfaces were dried in vacuum at 60 °C before FN coating and with nitrogen flow after FN coating. For static contact angles, 3 μL drops of Milli-Q water were deposited on the surface, each time in a new dry position. For dynamic contact angles, measurements were divided between advancing and receding. Advancing contact angles (ACA) were analyzed by gradually increasing the volume of the original sessile droplet (3 μL) until the solid–liquid–gas contact line began its expansion, whereas the receding contact angles (RCA) were obtained by gradually decreasing the volume of the droplet until the solid–liquid–gas contact line began to shrink. Both contact angles were calculated with a volume variation rate of 0.1 μL/s. Contact angle hysteresis (H) was also calculated, being the angle difference between ACA and RCA. Testing was performed nine times per each material and type of contact angle. The data correspond to the first cycle of advancing and receding. The total experiment time was less than 1 min and no hydration effect was observed during this time.

As the copolymers are hydrophobic and the interaction of FN with the surface of the materials is strong, no significant variations of FN characterization is expected in wet conditions in which the cell culture experiments were performed. In fact, we have previously demonstrated that FN network conformation is very similar in dry and wet conditions.^22^ Furthermore, cell adhesion and differentiation were analyzed with FBS free cell culture medium to avoid the effect of proteins other than FN adsorbed on surfaces.

### 2.3 Fibronectin Adsorption and Conformation

Overall FN availability as well as the exposure of the synergy binding sites on the materials were investigated by indirect enzyme-linked immunosorbent assay (ELISA). Samples were blocked in a DPBS/1%BSA solution (BB) and incubated with the primary rabbit polyclonal antibodies. To determine the availability of FN, rabbit polyclonal anti-FN antibody (1:10 000, Sigma-Aldrich) was incubated for 1 h, followed by 1 h incubation with biotinylated horse antirabbit secondary antibody (1:10 000, Vectorlabs), both at room temperature (RT). For the synergy domain samples were incubated with the mAb1937 antibody (1:20 000) (Sigma-Aldrich) in BB for 1 h at RT. Then, they were incubated with goat antimouse HRP-tagged secondary antibody (1:10 000) (Vectorlabs) in BB for 1 h at RT. After transferring the samples to another plate, HRP substrate reagent solution (A and B substrates, R&D Systems) was used for 20 min in the dark and finally reaction was stopped using stop solution (R&D Systems). Samples were washed several times with DPBS/0.5% Tween 20 between steps. Absorbance for both domains was measured at 450 nm (maximum absorbance) and 540 nm (background absorbance) with a Tecan Infinite M200 Pro (Switzerland) plate reader, using 3 replicates per composition for each domain.

### 2.4 Cell Culture Studies

Cell culture studies were performed with murine C2C12 myoblasts (Sigma-Aldrich), a cell line with short experimental times of cell differentiation. Cells were always thawed at least 2 days before the start of any assay, refreshing media the following day to the thawing. Cells were maintained between experiments in Dulbecco’s modified Eagle medium (DMEM, + 4.5 g/L d-glucose + l-glutamine, Gibco), with 1% penicillin/streptomycin (P/S, Gibco) and 20% fetal bovine serum (FBS, Gibco) at 37 °C and 5% CO_2_. Prior to seeding, copolymer samples were carefully sterilized by ultraviolet light for 30 min under the hood and then coated with FN as described previously.

For each assay, medium was removed from flasks (T75-T175) after expansion (always under 70% confluence) and washed with DPBS before adding trypsin/EDTA (Sigma-Aldrich) to force the detachment of the cells. After neutralizing the trypsin with growth medium (DMEM + 1% P/S + 20% FBS), cells were counted using a Neubauer chamber.

#### 2.4.1 Cell Adhesion Assays

To study the initial cell-material interactions, C2C12 cells were centrifuged (1000 rpm, 5 min), resuspended and seeded at 5000 cells/cm^2^ on the different FN coated surfaces with DMEM supplemented with 1% P/S without FBS for 3 h at 37 °C. Cells were fixed with 3.7% formaldehyde for 30 min at 4 °C and permeabilized with 0.1% Triton X-100 in DPBS for 10 min at room temperature. Samples were then blocked for 1 h at RT with blocking buffer (DPBS/1% BSA) and stained with primary antibody against mouse vinculin hVIN-1 (1:400) (Sigma-Aldrich) for another hour. Secondary antibody (Cy3 a-mouse) (1:200) was coupled with phalloidin (1:200) (Alexa Fluor-488) and incubated for 1 h at RT in the dark. Both antibodies were diluted in BB. Samples were washed several times with DPBS/0.5% Tween 20 between staining steps and finally mounted with VectaShield with DAPI (Vector Laboratories), responsible for the staining of the cellular nuclei. Image acquisition by different channels was carried out in an inverted Zeiss Axio Observer Z1 fluorescence microscope with oil immersion (×60) due to the small size of FAs. ImageJ software was used for image postprocessing.

#### 2.4.2 Myogenic Differentiation Assays

To analyze the influence of surface mobility on cell differentiation, cells were resuspended and cultured at a seeding density of 20 000 cells/cm^2^ on FN coated samples in the absence of FBS at 37 °C for 3 h. Once cells were correctly attached to the substrate, media was change to full differentiation conditions (DMEM + 1% P/S + 1% Insulin-Transferring-Selenium-X (ITS-X, Gibco)) and cultured for 4 days at 37 °C, refreshing media every 2 days. Part of the samples were cultured in the presence of blebbistatin at 10 μM, a contractility inhibitor of myosin II found in C2C12 cells myotube sarcomeres. Glass samples coated with collagen I at 1 mg/mL (Stemcell) were used as control.

After incubation, cells were fixed and permeabilized with 70% ethanol, 37% formaldehyde, and acid acetic glacial solution (20:2:1) at 4 °C for 10 min. After fixation, samples were rinsed with DPBS^–2^ several times and blocked with BB (DPBS/5% goat serum) for 1 h at room temperature. Samples were stained for sarcomeric myosin II by mouse primary antibody (1:250) (MF-20, Developmental Studies Hybridoma Bank) incubation in BB at 37 °C for 1 h followed by a blocking step of 30 min at room temperature, and incubation with secondary conjugated antibody (1:200) (Cy3 a-mouse) at 37 °C for 1 h in the dark. Samples were washed several times with DPBS/0.5% Tween 20 between staining steps. To conclude, samples (3 per each composition) were mounted on microscopy slides with VectaShield with DAPI and kept away from light until fluorescence image acquisition. Percentage of myogenic differentiation was calculated as the fraction between the number of nuclei found inside the stained myotubes and the total number of nuclei in each image using the Cellc12 program.^23^

### 2.5 Data Analysis

Data were statistically analyzed using GraphPad Prism 6 and reported as mean-standard deviation. D’Agostino-Pearson omnibus test was utilized to establish if data followed a normal distribution. One-way ANOVA tests were performed to determine any significant differences, *p ≤ 0.05, **p ≤ 0.01, ***p ≤ 0.001, and *p ≤ 0.0001, using Tukey HSD post hoc test for pairwise comparisons and nonparametric tests followed by a Dunn′s test in the contrary case.

## 3 Results and Discussion

**Figure 1:**
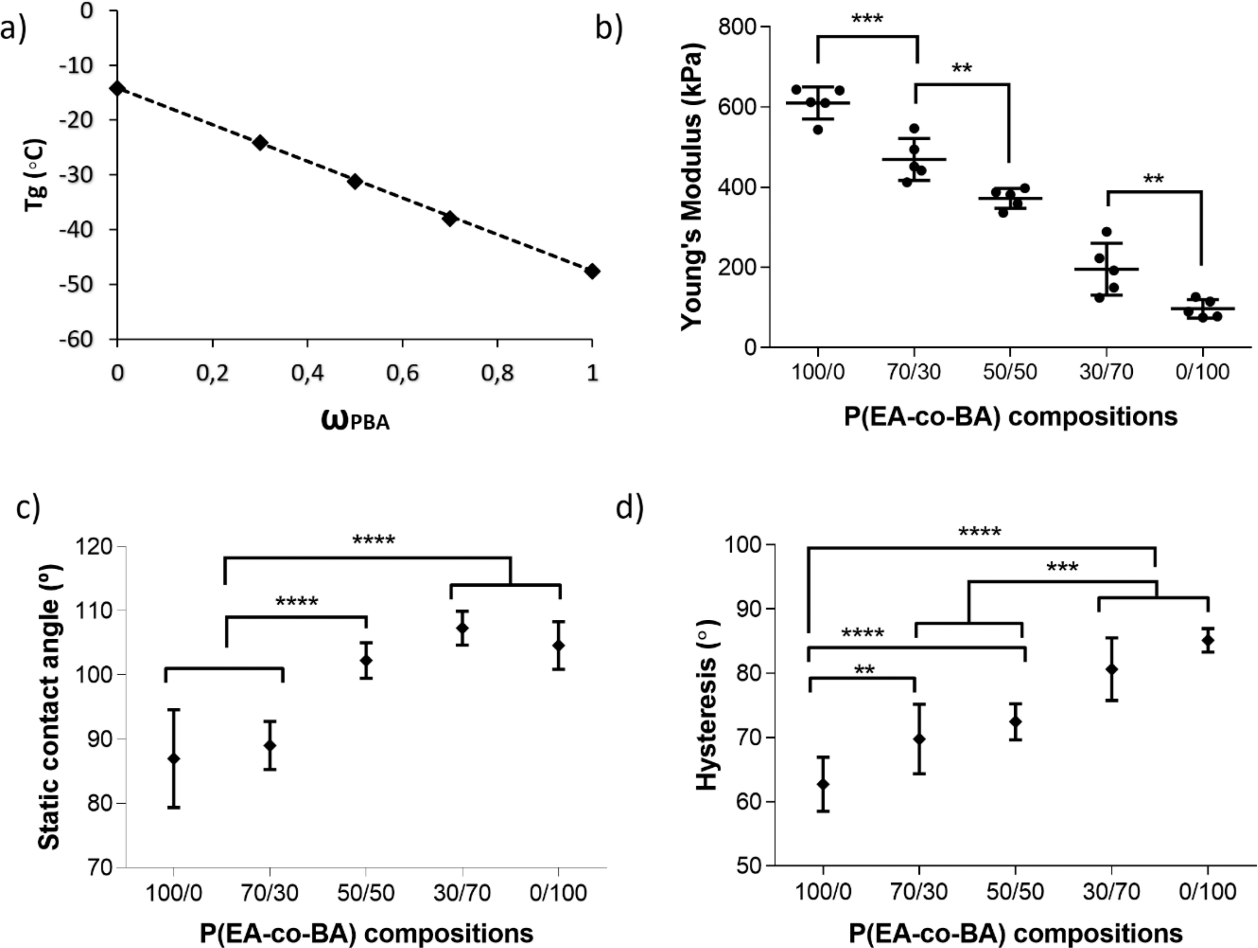
Characterization of the copolymer substrates. (a) Experimental glass transition temperature values (*T*_g_) and linear prediction for ideal mixtures. (b) Young’s moduli at uniaxial extension of bulk polymers. (c) Static contact angle values and (d) water contact angle hysteresis of spin-coated samples. In panel b, all compositions were significantly different from each other (*p* = ****) apart from the ones already shown.

As observed in Figure 1a, the glass transition of PEA is −14.2 °C and of PBA −47.5 °C; the copolymers following a linear tendency between the pure polymers probably due to the good miscibility between the polymers (a single glass transition was observed in the DSC scans, as observed in Figure S1). Although cell culture experiments were performed above the glass transition temperatures of the samples (at 37 °C), this difference on 30 degrees of the glass transition between PEA and PBA influences surface mobility, as other experiments in this study confirm. The macroscopic Young’s modulus at uniaxial extension of PEA (610 kPa) is approximately six times higher than that of PBA (96 kPa), the copolymers presenting intermediate values, all materials having significantly different values except between 50/50 and 30/70 (Figure 1b). Despite this significantly large difference, all samples possessed a Young’s modulus higher than 95 kPa, which is higher than the stiffness threshold of 40 kPa^24^ to which cells are sensitive to before feeling substrates as just “stiff”, and even greater than the Young’s modulus of skeletal muscle tissue that is 12 kPa.^25,26^ A similar result was previously obtained by nanoindentation, which resulted in Young’s modulus ≥1 MPa for PEA and PBA.^17^ Thus, all studied copolymers can be considered as stiff materials from the C2C12 perspective and mechanical properties influence on cell behavior can be neglected in this study. Stiff substrates are able to promote C2C12 adhesion and myogenic differentiation, which is characterized by elongated and thin myotubes. Contrarily, soft substrates allow differentiation only for few days with very short and thick myotubes.^27^ That is why our study was performed on stiff substrates, as they are optimal for myogenic differentiation.

Atomic force microscopy images of the surface of the materials (Figure S2) indicate that all the materials have a similar surface roughness (*R*_q_) (with average of 0.47 ± 0.15 nm) and are smooth. This means that topography cannot be considered as a possible cause of the different cell response on the surface of the materials. Reported studies demonstrate that low surface roughness and high stiffness promoted myogenic differentiation of C2C12 cells.^28^ That is the reason why we used smooth surfaces to analyze cell differentiation.

Static water contact angles (Figure 1c) present significant differences between PEA and PBA as well as between 50/50 and 30/70 copolymers, and show that copolymers become more hydrophobic as the content of PBA in them increases. Similar angle values between all conditions are obtained after coating the samples with fibronectin (Figure S3a). Water contact angle hysteresis of Figure 1d significantly increases with the PBA content of the copolymers due to the gradual increase of the advancing angle but similar receding angle. As previously reported, hysteresis is an indication of surface mobility^29,30^ denoting a higher surface mobility in PBA than PEA, with increasing values on the copolymers with higher PBA content. When samples were coated with FN, hysteresis increased in all the compositions (Figure S3) due to an increase in the advancing angle and a decrease of the receding angle (Figure S3). The increase in hysteresis may correlate with an enhanced ability of adsorbed FN molecules to carry out rearrangement at the water/air interface,^17^ which can impact cell behavior.

**Figure 2:**
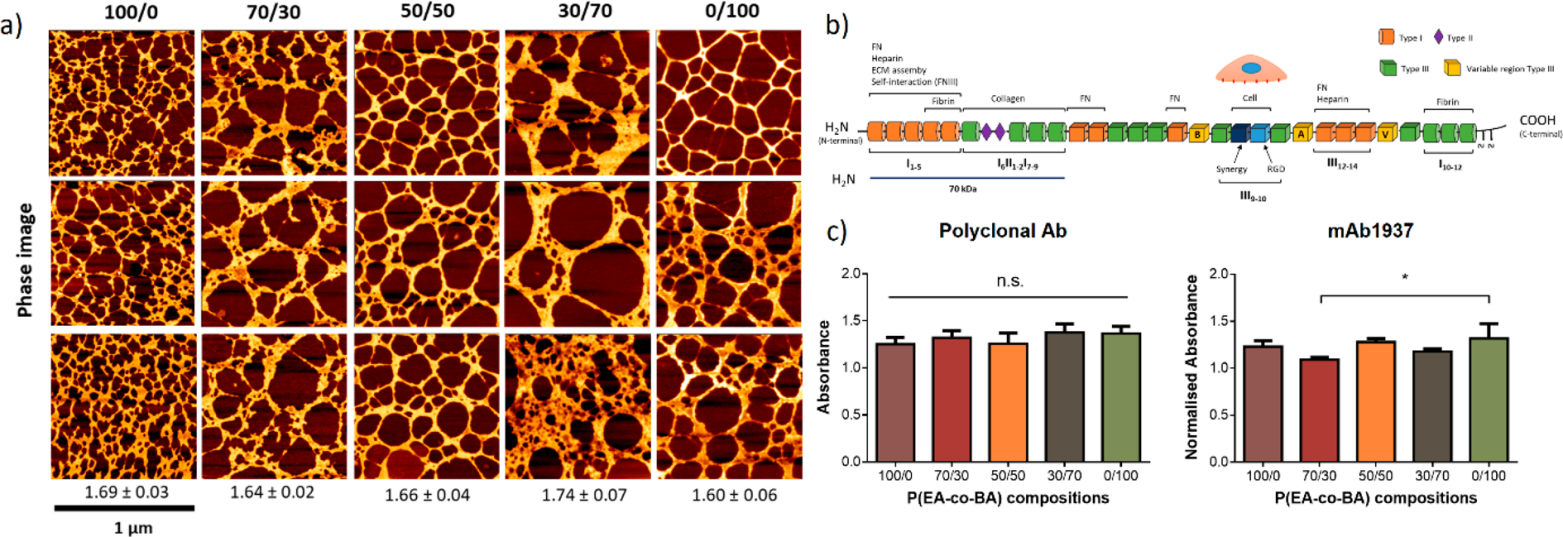
FN absorption and conformation after 1 h of incubation at 20 μg mL^–1^. (a) 1 μm × 1 μm AFM images (phase magnitude) of FN distribution after adsorption in the different substrates. Fractal dimension (FD) values and standard deviations are shown below for each composition. (b) Schematic structure of fibronectin (FN) and its binding domains. FN consists of three types of reiterating modules (type I, type II, and type III), which are organized into specific domains and can interact with multiple binding partners, such as other FN dimers (orange), heparin, collagen, fibrin, or cell-matrix adhesion receptors (blue), as indicated. The three alternatively spliced type III segments EIIIB (B), EIIIA (A), and IIICS (V) (yellow) generate the two main forms of FN, cellular and plasma FN. (c) Surface exposition of FN available binding domains using anti-FN polyclonal antibody (left) and PHSRN synergy domain (FNIII9) (right). Monoclonal antibody mAb1937 against this domain of FN was used.

As observed in Figure 2a, the conformation of FN on the copolymers is fibrillar regardless the EA/BA ratio, which means that material-driven fibrillogenesis is produced not only in the pure systems (as previously demonstrated^17^) but also in all the copolymers. None of the compositions promotes a globular conformation of the protein. To study the network complexity and degree of fibrillogenesis we calculated the fractal dimension that is indicated by the numbers below the pictures in Figure 2a and the graph in Figure S4b. Fractal dimension values are similar for PEA, 70/30 and 50/50, whereas we found significant differences between 30/70 and PBA. The 30/70 network seems to be thicker in the AFM images, which could be the cause of the higher fractal dimension. This suggests that the incorporation of small concentrations of PEA to PBA is able to induce more interconnected FN nanonetworks than in the rest of copolymers. PBA has the lowest value of the fractal dimension, suggesting a decrease in the FN network connections.

Material-driven FN fibrillogenesis is at first a process mediated by the exposure of specific FN-FN domains, which causes interaction between unfolded FN molecules to form fibrils.^4^ Moreover, the exposure of other available binding sites is also vital for cell adhesion through integrin-receptor interactions. General availability of FN and exposure of the synergy domain observed in Figure 2b were almost the same for all the compositions, except the 70/30 sample that has slightly lower exposure of the synergy domain. This indicates that FN conformation is very similar in all the copolymers and is not a determinant parameter in cell behavior on the biomaterials.

**Figure 3:**
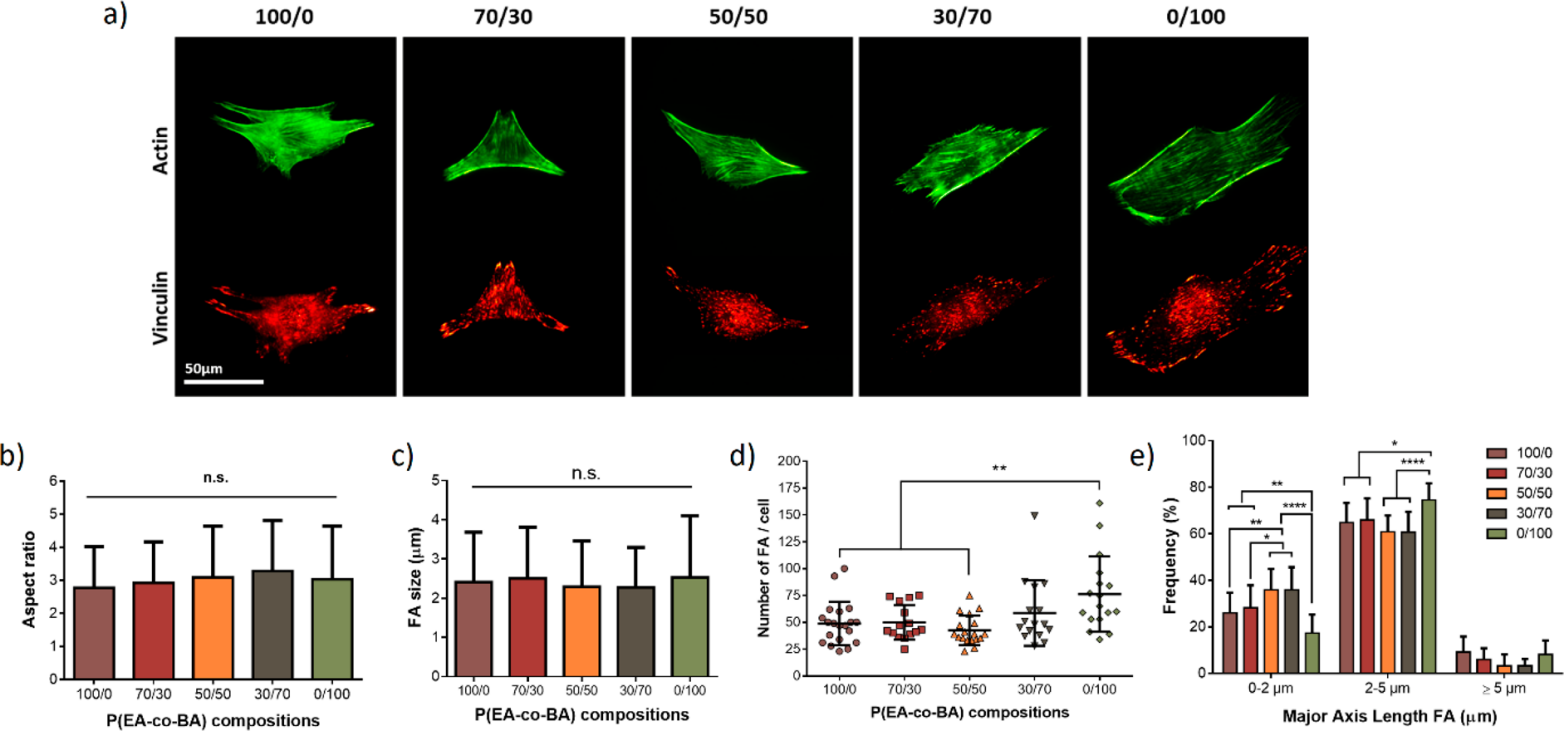
Cell adhesion and morphology after 3 h of culture on FN coated substrates. (a) Immunofluorescence for cell actin cytoskeleton (green) and vinculin protein (red) for focal adhesions (FA). (b) Cell aspect ratio, (c) mean FA values, (d) number of FAs per cell and composition and (e) grouped frequency distribution of FA major axis length. Aspect ratio was calculated as the ratio between cell major axis and cell minor axis.

C2C12 cell response on P(EA-*co*-BA) copolymers was studied, analyzing cell morphology, focal adhesions and myogenic differentiation with the aim of studying how surface mobility influences cell response, as this parameter was found to change among the different compositions. Cells are well spread on all the samples with a very well developed actin cytoskeleton, as seen in Figure 3a.

The aspect ratio represented in Figure 3b is the resultant ratio between the cell length and width,^12^ where circular shapes are near to one while elongated forms present higher values. The cells in all the materials are elongated, with no significant differences in the aspect ratio among the different copolymers, reaching values close to 3, a value previously reported for C2C12 cultured on gelatin-based films.^31^

Focal adhesions (FA) were analyzed after 4 h of cell culture by staining the vinculin protein (images of Figure 3a). The majority of FAs are located at the edge of the cells. In general, FA size is similar in all the compositions (Figure 3c) with no significant differences among the samples. However, the number of FA (Figure 3d) is significantly higher for the PBA sample, as already remarked in previous studies where PBA had more focal adhesions formed than PEA.^1^ To deeper analyze the influence of surface mobility on FA formation, the distribution of FA sizes was represented in Figure 3e. In the range up to 0–2 μm (representing immature focal adhesions) PBA has the lowest frequency of FAs, followed by PEA and 70/30, which had a lower frequency than the rest of copolymers. This result shows that focal adhesions in the copolymers tend to be of smaller size than in pure materials. In the range of medium size, between 2 and 5 μm corresponding to maturing focal adhesions plaques, PBA presents a higher frequency than PEA and 70/30, having the other copolymers 50/50 and 30/70 significant lower frequencies than PBA. A similar result is observed for mature FA size ≥5 μm in which the pure components demonstrate the higher frequency and the copolymers the lower. All these results demonstrate that the cells within the materials are well adhered, mainly forming focal adhesions of the size in the range of 2–5 μm and that the change in the interfacial mobility had a slight influence on the size of FA that is lower in the copolymers than in the pure materials.

**Figure 4:**
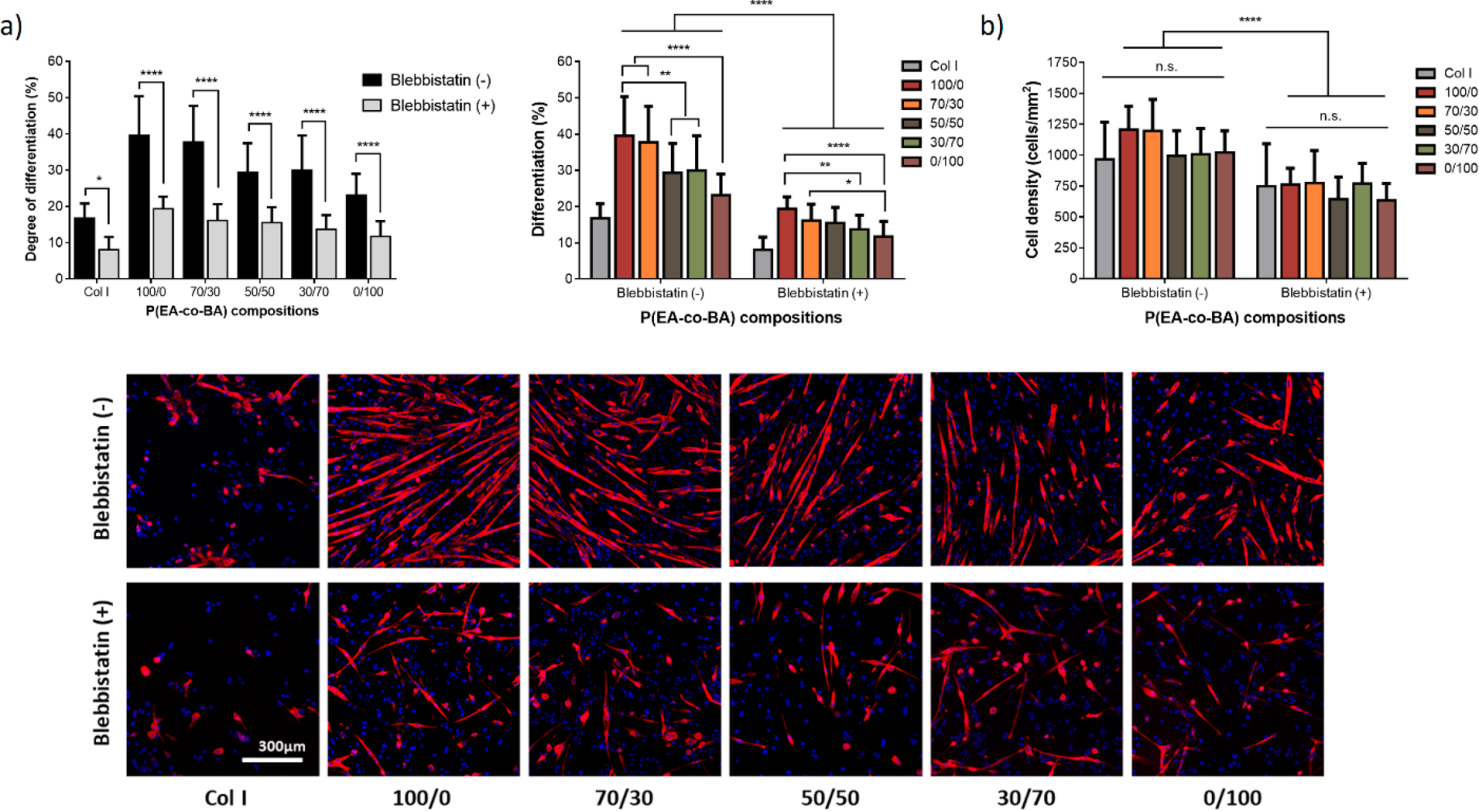
Myogenic differentiation of C2C12 cells after 4 days of culture. Cells were cultured on FN coated collagen I (control sample) and the different copolymer surfaces with and without blebbistatin (10 μM), which is used as a cell contractility inhibitor. (a) Percentages of cell differentiation measured as the fraction of cells situated inside the sarcomeric myotubes. (b) Cell densities with and without blebbistatin. The images below correspond to the immunofluorescence of sarcomeric myosin (red) and cell nuclei (blue) for each of the polymer surfaces. Col I differentiation degree was found significantly different in each group from 4 other compositions with *p* ≤ 0.01.

Images in Figure 4 show cell nuclei in blue and sarcomeric myosin stained in red in normal cell differentiation medium and in the presence of blebbistatin that is used as contractility inhibitor. Cells in all the samples express sarcomeric myosin and in higher amount as the positive control do, collagen type I. As expected, cell differentiation is higher in the absence of blebbistatin, which demonstrates that C2C12 cells need to activate their contractility for differentiation (Figure 4a).^32^ In both conditions, with and without blebbistatin, cell differentiation is higher in PEA than in PBA, the copolymers 50/50 and 30/70 show significant lower degrees of cell differentiation than pure PEA. This same trend is seen when the contractility of cells is inhibited. Cell density is almost equal in all the compositions (Figure 4b). Thus, as the surface mobility increases, cell differentiation diminishes without any intermediate copolymer having better values than the PEA polymer. This study demonstrates that even though the cells on the copolymers had smaller focal adhesions, this did not affect cell differentiation and molecular mobility of the surface was the only parameter regulating cell differentiation. The higher the mobility of the surface, the lower the degree of the resulting cell differentiation.

## 4 Conclusions

This study has demonstrated that surface mobility of biomaterials can be regulated by the synthesis of copolymers of PEA and PBA in the whole range of compositions. PEA possessing the lowest surface mobility and this gradually increasing with increasing concentrations of PBA. All the resulting copolymers produced from a combination of these two polymers induce the organization of fibronectin on their surface in the form of nanonetworks with a similar structure, demonstrated by the similar availability of the domains of FN. Surface mobility did not influence cell morphology on the substrates, despite the frequency of smaller foal adhesions being larger on the copolymer surfaces than in pure polymers. Here we show cell differentiation being regulated by surface mobility in a controlled and regulated way, PEA inducing the highest degree of C2C12 myoblast differentiation, followed by PBA copolymers and ultimately PBA. With these results, we confirm that as the surface mobility is lower, the percentage of differentiated contractile cells is higher.

## Supporting Information Available

Differential scanning calorimetry scans, the height images of the atomic force microscopy with the obtained root-mean square roughness of the samples, details about the static and dynamic contact angle measurements of the samples without and with fibronectin coating, and a graph with the fractal dimension of fibronectin network on the surface of the copolymers (PDF)

## Abbreviations

ACA: advancing contact angles
AFM: atomic force microscopy
BA: butyl acrylate
BB: blocking buffer solution of DPBS/1%BSA
BCM: box-counting method
DMEM: Dulbecco’s modified Eagle medium
DPBS: Dulbecco’s Phosphate Saline Buffer
DSC: differential scanning calorimeter
*E*: Young’s modulus
EA: ethyl acrylate
ELISA: indirect enzyme-linked immunosorbent assay
FA: focal adhesions
FD: fractal dimension
FN: fibronectin
*H*: contact angle hysteresis
ITS-X: Insulin-Transferring-Selenium-X
PBA: poly(buthyl acrylate)
PEA: poly(ethyl acrylate)
PHA: poly(hexyl acrylate)
PMA: poly(methyl acrylate)
RCA: receding contact angles
RMS: root-mean-square
*R*_q_: surface roughness
RT: room temperature
*T*_g,_: glass transition temperature
WCA: water contact angle

## References

[ref1] González-García C.; Moratal D.; Oreffo R. O. C.; Dalby M. J.; Salmerón-Sánchez M. Surface Mobility Regulates Skeletal Stem Cell Differentiation. Integr. Biol. 2012, 4, 531–539. 10.1039/c2ib00139j. 22395101

[ref2] García A. J. Get a Grip: Integrins in Cell-Biomaterial Interactions. Biomaterials 2005, 26, 7525–7529. 10.1016/j.biomaterials.2005.05.029. 16002137

[ref3] Dalby M. J.; García A. J.; Salmeron-Sanchez M. Receptor Control in Mesenchymal Stem Cell Engineering. Nat. Rev. Mater. 2018, 3, 17091 10.1038/natrevmats.2017.91.

[ref4] Salmerón-Sánchez M.; Rico P.; Moratal D.; Lee T. T.; Schwarzbauer J. E.; García A. J. Role of Material-Driven Fibronectin Fibrillogenesis in Cell Differentiation. Biomaterials 2011, 32, 2099–2105. 10.1016/j.biomaterials.2010.11.057. 21185593

[ref5] Rico P.; Hernández J. C. R.; Moratal D.; Altankov G.; Pradas M. M.; Salmerón-Sánchez M. Substrate-Induced Assembly of Fibronectin into Networks: Influence of Surface Chemistry and Effect on Osteoblast Adhesion. Tissue Eng. - Part A 2009, 15, 3271–3281. 10.1089/ten.tea.2009.0141. 19382854

[ref6] Llopis-Hernández V.; Cantini M.; González-García C.; Cheng Z. A.; Yang J.; Tsimbouri P. M.; García A. J.; Dalby M. J.; Salmerón-Sánchez M. Material-Driven Fibronectin Assembly for High-Efficiency Presentation of Growth Factors. Sci. Adv. 2016, 2, e1600188 10.1126/sciadv.1600188. 27574702 PMC5001810

[ref7] Moulisová V.; Gonzalez-García C.; Cantini M.; Rodrigo-Navarro A.; Weaver J.; Costell M.; Sabater i Serra R.; Dalby M. J.; García A. J.; Salmerón-Sánchez M. Engineered Microenvironments for Synergistic VEGF – Integrin Signalling during Vascularization. Biomaterials 2017, 126, 61–74. 10.1016/j.biomaterials.2017.02.024. 28279265 PMC5354119

[ref8] Cheng Z. A.; Alba-Perez A.; Gonzalez-Garcia C.; Donnelly H.; Llopis-Hernandez V.; Jayawarna V.; Childs P.; Shields D. W.; Cantini M.; Ruiz-Cantu L.; Reid A.; Windmill J. F. C.; Addison E. S.; Corr S.; Marshall W. G.; Dalby M. J.; Salmeron-Sanchez M. Nanoscale Coatings for Ultralow Dose BMP-2-Driven Regeneration of Critical-Sized Bone Defects. Adv. Sci. 2019, 6, 1800361 10.1002/advs.201800361. PMC6343071 30693176

[ref9] Vanterpool F. A.; Cantini M.; Seib F. P.; Salmerón-Sánchez M. A Material-Based Platform to Modulate Fibronectin Activity and Focal Adhesion Assembly. Biores. Open Access 2014, 3, 286–296. 10.1089/biores.2014.0033. 25469314 PMC4245838

[ref10] González-García C.; Cantini M.; Ballester-Beltrán J.; Altankov G.; Salmerón-Sánchez M. The Strength of the Protein-Material Interaction Determines Cell Fate. Acta Biomater. 2018, 77, 74–84. 10.1016/j.actbio.2018.07.016. 30006313

[ref11] Seo J. H.; Yui N. The Effect of Molecular Mobility of Supramolecular Polymer Surfaces on Fibroblast Adhesion. Biomaterials. 2013, 34, 55–63. 10.1016/j.biomaterials.2012.09.063. 23079667

[ref12] Arisaka Y.; Yui N. Polyrotaxane-Based Biointerfaces with Dynamic Biomaterial Functions. J. Mater. Chem. B 2019, 7, 2123–2129. 10.1039/C9TB00256A. 32073570

[ref13] Seo J.-H.; Hirata M.; Kakinoki S.; Yamaoka T.; Yui N. Dynamic Polyrotaxane-Coated Surface for Effective Differentiation of Mouse Induced Pluripotent Stem Cells into Cardiomyocytes. RSC Adv. 2016, 6, 35668–35676. 10.1039/C6RA03967G.

[ref14] Arisaka Y.; Masuda H.; Yoda T.; Yui N. Delayed Senescence of Human Vascular Endothelial Cells by Molecular Mobility of Supramolecular Biointerfaces. Macromol. Biosci. 2021, 21, 2100216 10.1002/mabi.202100216. 34390172

[ref15] Kakinoki S.; Seo J. H.; Inoue Y.; Ishihara K.; Yui N.; Yamaoka T. Mobility of the Arg-Gly-Asp Ligand on the Outermost Surface of Biomaterials Suppresses Integrin-Mediated Mechanotransduction and Subsequent Cell Functions. Acta Biomater. 2015, 13, 42–51. 10.1016/j.actbio.2014.11.020. 25463493

[ref16] Roth C. B.; Dutcher J. R. Glass Transition and Chain Mobility in Thin Polymer Films. J. Electroanal. Chem. 2005, 584, 13–22. 10.1016/j.jelechem.2004.03.003.

[ref17] Bathawab F.; Bennett M.; Cantini M.; Reboud J.; Dalby M. J.; Salmerón-Sánchez M. Lateral Chain Length in Polyalkyl Acrylates Determines the Mobility of Fibronectin at the Cell/Material Interface. Langmuir. 2016, 32, 800–809. 10.1021/acs.langmuir.5b03259. 26715432 PMC4732669

[ref18] Mnatsakanyan H.; Rico P.; Grigoriou E.; Candelas A. M.; Rodrigo-Navarro A.; Salmeron-Sanchez M.; Sabater i Serra R. Controlled Assembly of Fibronectin Nanofibrils Triggered by Random Copolymer Chemistry. ACS Appl. Mater. Interfaces. 2015, 7, 18125–18135. 10.1021/acsami.5b05466. 26225535

[ref19] Guerra N. B.; González-García C.; Llopis V.; Rodríguez-Hernández J. C.; Moratal D.; Rico P.; Salmerón-Sánchez M. Subtle Variations in Polymer Chemistry Modulate Substrate Stiffness and Fibronectin Activity. Soft Matter. 2010, 6, 4748–4755. 10.1039/c0sm00074d.

[ref20] Kourouklis A. P.; Lerum R. V.; Bermudez H. Cell Adhesion Mechanisms on Laterally Mobile Polymer Films. Biomaterials. 2014, 35, 4827–4834. 10.1016/j.biomaterials.2014.02.052. 24651034

[ref21] Sekiya-Aoyama R.; Arisaka Y.; Yui N. Mobility Tuning of Polyrotaxane Surfaces to Stimulate Myocyte Differentiation. Macromol. Biosci. 2020, 20, 1900424 10.1002/mabi.201900424. 32058659

[ref22] Sprott M. R.; Gallego-Ferrer G.; Dalby M. J.; Salmerón-Sánchez M.; Cantini M. Functionalization of PLLA with Polymer Brushes to Trigger the Assembly of Fibronectin into Nanonetworks. Adv. Healthc. Mater. 2019, 8, e1801469 10.1002/adhm.201801469. 30609243

[ref23] Selinummi J.; Seppälä J.; Yli-Harja O.; Puhakka J. A. Software for Quantification of Labeled Bacteria from Digital Microscope Images by Automated Image Analysis. Biotechniques. 2005, 39, 859–862. 10.2144/000112018. 16382904

[ref24] Balaban N. Q.; Schwarz U. S.; Riveline D.; Goichberg P.; Tzur G.; Sabanay I.; Mahalu D.; Safran S.; Bershadsky A.; Addadi L.; Geiger B. Force and Focal Adhesion Assembly: A Close Relationship Studied Using Elastic Micropatterned Substrates. Nat. Cell Biol. 2001, 3, 466–472. 10.1038/35074532. 11331874

[ref25] Gilbert P. M.; Havenstrite K. L.; Magnusson K. E. G.; Sacco A.; Leonardi N. A.; Kraft P.; Nguyen N. K.; Thrun S.; Lutolf M. P.; Blau H. M. Substrate Elasticity Regulates Skeletal Muscle Stem Cell Self-Renewal in Culture. Science. 2010, 329, 1078–1081. 10.1126/science.1191035. 20647425 PMC2929271

[ref26] Hayashi K.; Matsuda M.; Mitake N.; Nakahata M.; Munding N.; Harada A.; Kaufmann S.; Takashima Y.; Tanaka M. One-Step Synthesis of Gelatin-Conjugated Supramolecular Hydrogels for Dynamic Regulation of Adhesion Contact and Morphology of Myoblasts. ACS Appl. Polym. Mater. 2022, 4, 2595–2603. 10.1021/acsapm.1c01902.

[ref27] Ren K.; Crouzier T.; Roy C.; Picart C. Polyelectrolyte Multilayer Films of Controlled Stiffness Modulate Myoblast Cell Differentiation. Adv. Funct. Mater. 2008, 18, 1378–1389. 10.1002/adfm.200701297. 18841249 PMC2561337

[ref28] Hu X.; Park S. H.; Gil E. S.; Xia X. X.; Weiss A. S.; Kaplan D. L. The influence of elasticity and surface roughness on myogenic and osteogenic-differentiation of cells on silk-elastin biomaterials. Biomaterials. 2011, 32, 8979–89. 10.1016/j.biomaterials.2011.08.037. 21872326 PMC3206257

[ref29] Van Damme H. S.; Hogt A. H.; Feijen J. Surface Mobility and Structural Transitions of Poly(n-Alkyl Methacrylates) Probed by Dynamic Contact Angle Measurements. J. Colloid Interface Sci. 1986, 114, 167–172. 10.1016/0021-9797(86)90248-1.

[ref30] Takahashi S.; Kasemura T.; Asano K. Surface Molecular Mobility for Copolymers Having Perfluorooctyl and/or Polyether Side Chains via Dynamic Contact Angle. Polymer. 1997, 38, 2107–2111. 10.1016/S0032-3861(96)00770-7.

[ref31] Grover C. N.; Farndale R. W.; Best S. M.; Cameron R. E. The Interplay between Physical and Chemical Properties of Protein Films Affects Their Bioactivity. J. Biomed. Mater. Res. Part A 2012, 100A, 2401–2411. 10.1002/jbm.a.34187. 22528690

[ref32] Allingham J. S.; Smith R.; Rayment I. The Structural Basis of Blebbistatin Inhibition and Specificity for Myosin II. Nat. Struct. Mol. Biol. 2005, 12, 378–379. 10.1038/nsmb908. 15750603

