# Supplementary material for: Fine-Tuning Regulation of Surface Mobility by Acrylate Copolymers and Its Effect on Cell Adhesion and Differentiation
Source: ACS Appl Bio Mater. 2023 Apr 17;6(5):1755–62. doi: 10.1021/acsabm.2c01053 (PMC10189726; doi:10.1021/acsabm.2c01053)
Supplement: Supplementary file 1 — mt2c01053_si_001.pdf [file mt2c01053_si_001.pdf]

SUPPORTING INFORMATION

Fine-tuning regulation of surface mobility by acrylate copolymers and its effect on cell adhesion and differentiation

Miranda Morata-Martínez[a,b], Mark R. Sprott[b], Carmen M. Antolinos-Turpín[a], Manuel Salmeron-Sanchez[a,b,c,], and Gloria Gallego-Ferrer[a c,*]*

[a] Centre for Biomaterials and Tissue Engineering (CBIT), Universitat Politècnica de València, 46022 Valencia, Spain.

[b] Centre for the Cellular Microenvironment, University of Glasgow, G12 8LT, Glasgow, United Kingdom.

[c] Biomedical Research Networking Center on Bioengineering, Biomaterials and Nanomedicine (CIBER-BBN), 46022 Valencia, Spain.

Corresponding Authors

* Manuel Salmeron-Sanchez - Centre for Biomaterials and Tissue Engineering (CBIT), Universitat Politècnica de València, 46022 Valencia, Spain; Centre for the Cellular Microenvironment, University of Glasgow, G12 8LT, Glasgow, United Kingdom; Biomedical Research Networking Center on Bioengineering, Biomaterials and Nanomedicine (CIBER-BBN), 46022 Valencia, Spain.; ORCID: 0000-0002-8112-2100; E-mail: Manuel.Salmeron-Sanchez@glasgow.ac.uk

* Gloria Gallego-Ferrer - Centre for Biomaterials and Tissue Engineering (CBIT), Universitat Politècnica de València, 46022 Valencia, Spain; Biomedical Research Networking Center on Bioengineering, Biomaterials and Nanomedicine (CIBER-BBN), 46022 Valencia, Spain.; ORCID: 0000-0002-2428-0903; E-mail: ggallego@ter.upv.es

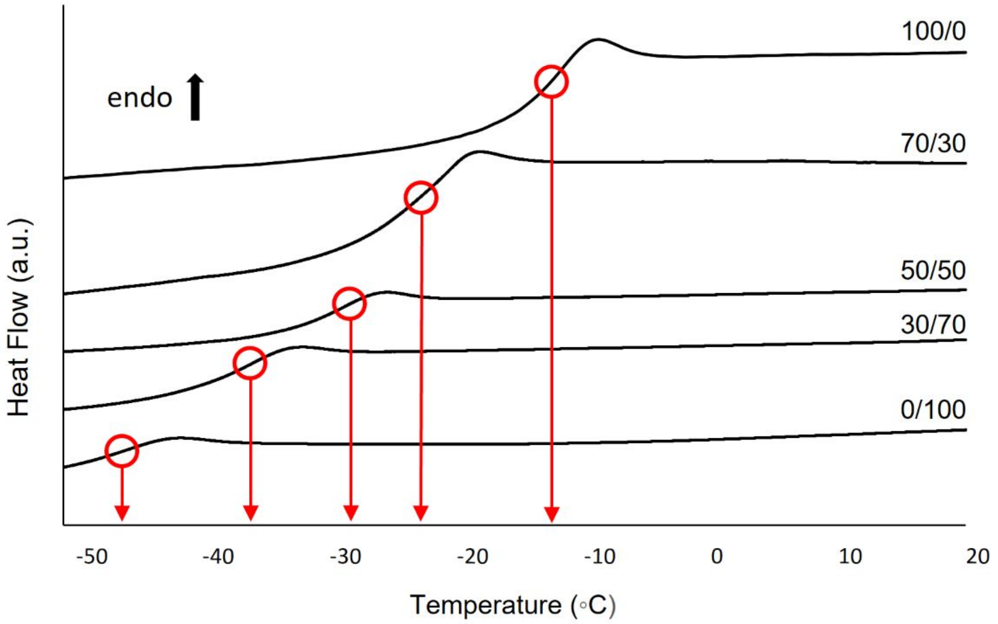

Figure S1. Differential scanning calorimetry (DSC) scans for the second heating. The red circles indicate the glass transition temperature of the different copolymers calculated as the midpoint of the change in the specific heat capacity. Heat flow units are arbitrary units to prevent scans overlapping.

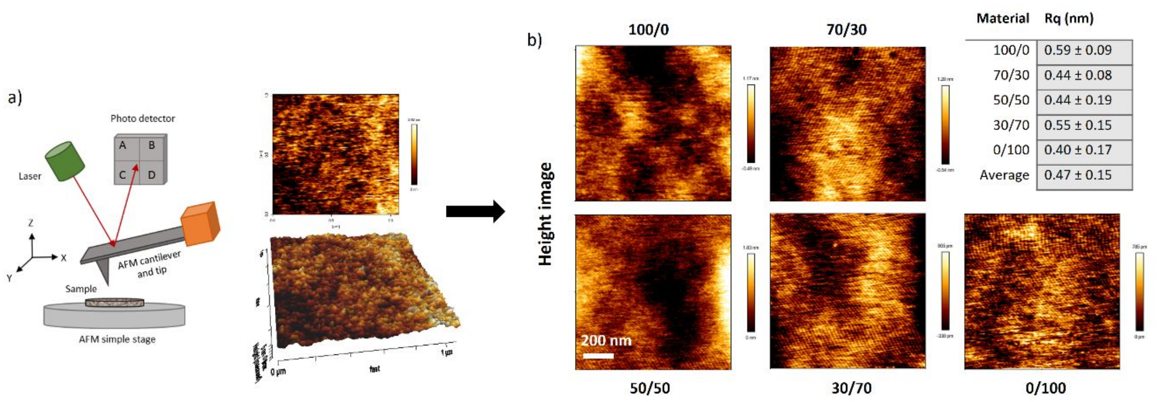

Figure S2. Analysis of samples topography by AFM. a) Diagram of an AFM working system. 3D surface representation from 2D data acquisition. b) 1 μm x 1 μm height images of the different copolymers surfaces were obtained to measure root-mean-square roughness (Rq).

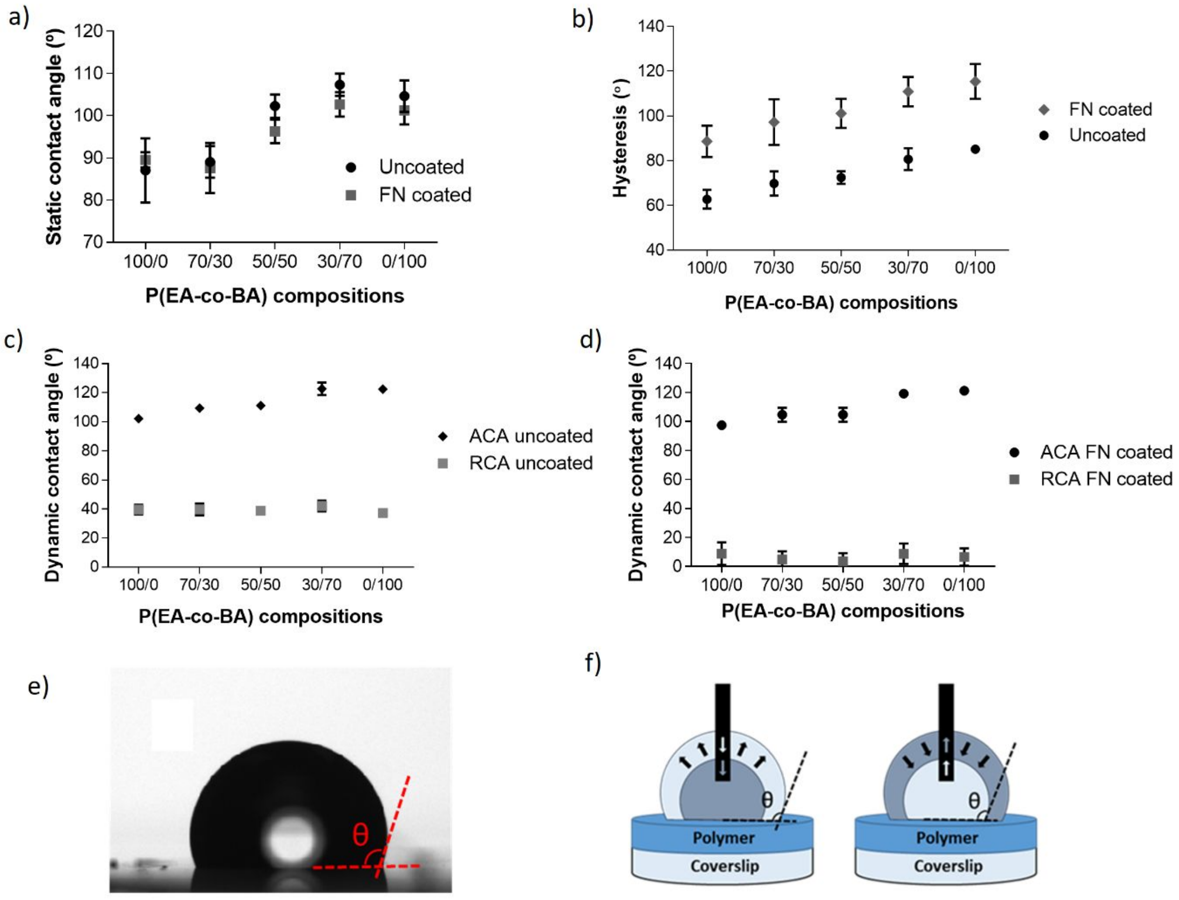

Figure S3. Copolymer surface wettability analysis before and after 1 h of FN coating with 20 µg/mL. a) Static water contact angle (WCA) values before coating and with FN coating. b) WCA hysteresis values before and after coating on surface polymers. c) Dynamic contact angles, advancing (ACA) and receding (RCA), before and d) after coating. e) Representation of a WCA (red) reading on the liquid-solid-gaseous interface, using a tensiometer. Since the contact angle θ is higher than 90°, the substrate in question is considered hydrophobic. (f) Dynamic contact angles reading, advancing angle (left) and receding angle (right).

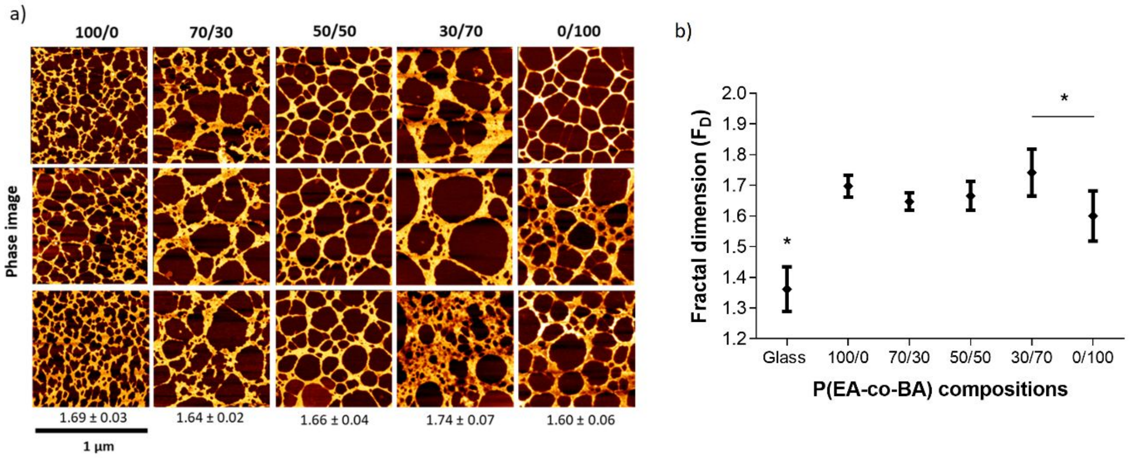

Figure S4. FN absorption and conformation after 1 h incubation at 20 µg mL^{-1}. a) 1µm x 1µm AFM images (phase magnitude) of FN distribution after adsorption in the different substrates. b) Fractal dimension (F_D) values for each composition. Values from globular FN on glass are also presented for comparison.
